# Immunosuppression in adult liver transplant recipients: a 2024 update from the Italian Liver Transplant Working Group

**DOI:** 10.1007/s12072-024-10703-4

**Published:** 2024-07-15

**Authors:** Tommaso Maria Manzia, Barbara Antonelli, Amedeo Carraro, Grazia Conte, Nicola Guglielmo, Andrea Lauterio, Laura Mameli, Umberto Cillo, Luciano De Carlis, Massimo Del Gaudio, Paolo De Simone, Stefano Fagiuoli, Francesco Lupo, Giuseppe Tisone, Riccardo Volpes, on behalf of the Italian Liver Transplant Working Group

**Affiliations:** 1 Department of Surgical Science, University of Rome Tor Vergata, Rome, Italy; 2 Fondazione IRCCS Ca’ Granda Ospedale Maggiore Policlinico, Milan, Italy; 3 Liver Transplant Unit, University Hospital Trust of Verona, Verona, Italy; 4 Clinica di Chirurgia Epatobiliare, Pancreatica e dei Trapianti, Azienda Ospedaliera Universitaria delle Marche, Ancona, Italy; 5 General Surgery and Liver Transplantation Unit, Azienda Ospedaliera San Camillo-Forlanini, Rome, Italy; 6 ASST Grande Ospedale Metropolitano Niguarda, University of Milano-Bicocca, Milan, Italy; 7 Azienda Ospedaliera G. Brotzu, Cagliari, Italy; 8 Hepatobiliary and Liver Transplant Unit, University Hospital of Padua, Padua, Italy; 9 Department of General Surgery and Transplantation, Niguarda Hospital, Milan, Italy; 10 School of Medicine, University of Milano-Bicocca, Milan, Italy; 11 Department of General Surgery and Transplantation, Policlinico S. Orsola-Malpighi, Bologna, Italy; 12 Hepatobiliary Surgery and Liver Transplantation Unit, University of Pisa Medical School Hospital, Pisa, Italy; 13 Gastroenterology, Department of Medicine, University of Milano-Bicocca and Gastroenterology Hepatology and Transplantation, Papa Giovanni XXIII Hospital, Piazza OMS, 124127 Bergamo, Italy; 14 Department of General Surgery, Azienda Ospedaliera Città Della Salute e Della Scienza, Turin, Italy; 15 Mediterranean Institute for Transplantation and Advanced Specialized Therapies (ISMETT/IRCCS), Palermo, Italy; 16 Fondazione Istituto G. Giglio di Cefalù, Palermo, Italy

**Keywords:** Calcineurin, Hepatocellular cancer, Immunosuppression, Liver transplantation, Liver metastasis, mTOR inhibitor, Nephrotoxicity, Rejection, Recurrence

## Abstract

**Purpose:**

Advances in surgical procedures and immunosuppressive therapies have considerably improved the outcomes of patients who have undergone liver transplantation in the past few decades. In 2020, the Italian Liver Transplant Working Group published practice-oriented algorithms for immunosuppressive therapy (IT) in adult liver transplant (LT) recipients. Due to the rapidly evolving LT field, regular updates to the recommendations are required. This review presents a consensus- and evidence-based update of the 2020 recommendations.

**Methods:**

The Italian Liver Transplant Working Group set out to address new IT issues, which were discussed based on supporting literature and the specialists’ personal experiences. The panel deliberated on and graded each statement before consensus was reached.

**Results:**

A series of consensus statements were formulated and finalized on: (i) oncologic indications for LT; (ii) management of chronic LT rejection; (iii) combined liver–kidney transplantation; (iv) immunosuppression for transplantation with an organ donated after circulatory death; (v) transplantation in the presence of frailty and sarcopenia; and (vi) ABO blood group incompatibility between donor and recipient. Algorithms were updated in the following LT groups: standard patients, critical patients, oncology patients, patients with specific etiology, and patients at high immunologic risk. A steroid-free approach was generally recommended, except for patients with autoimmune liver disease and those at high immunologic risk.

**Conclusion:**

The updated consensus- and evidence-based 2024 recommendations for immunosuppression regimens in adult patients with ABO-compatible LT address a range of clinical variables that should be considered to optimize the choice of the immunosuppression treatment in clinical practice in Italy.

**Graphical abstract:**

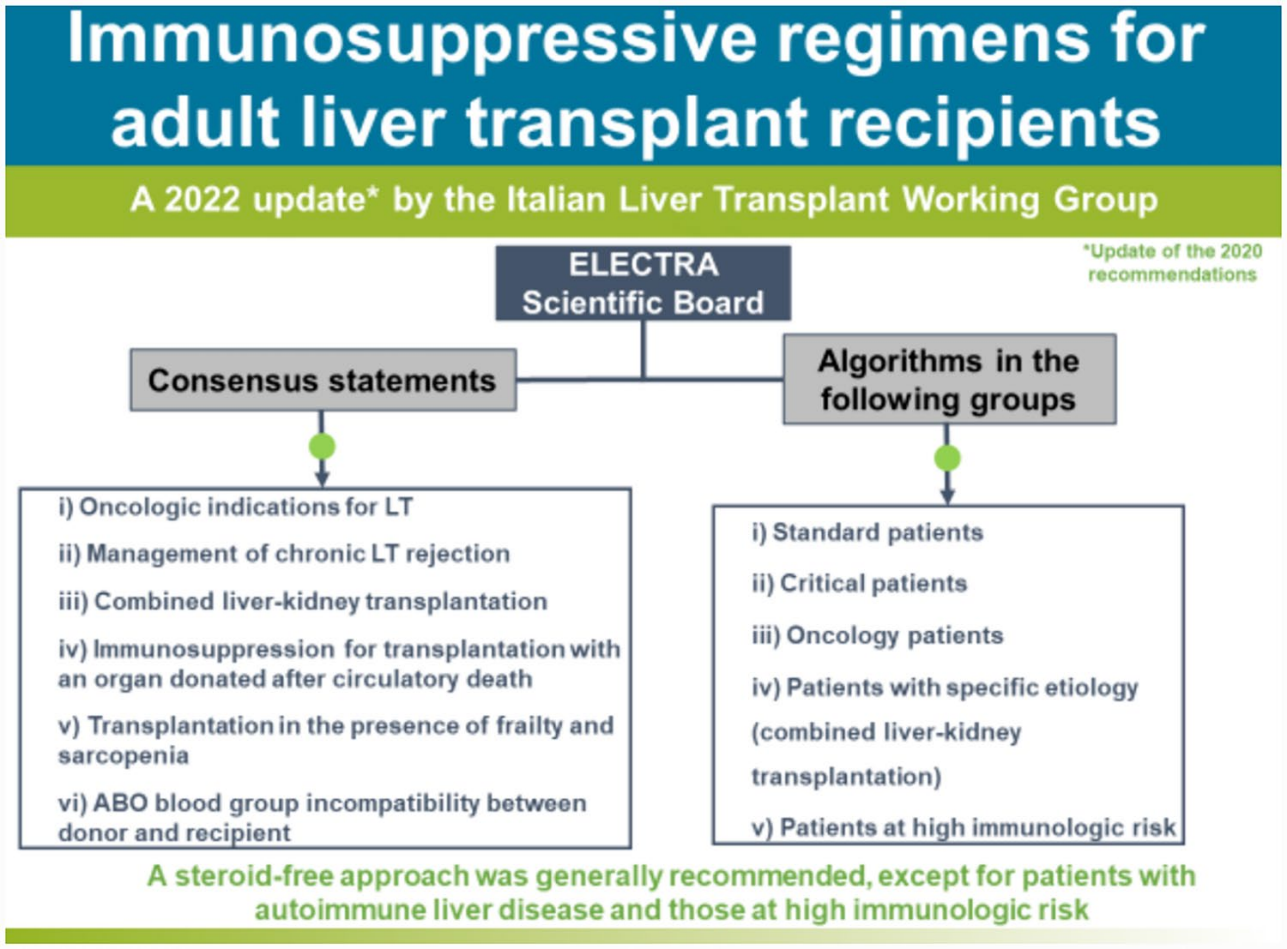

**Supplementary Information:**

The online version contains supplementary material available at 10.1007/s12072-024-10703-4.

## Introduction

Advances in surgical procedures and immunosuppressive therapy have substantially contributed to improving the outcomes of liver transplantation. Over the past few decades, calcineurin inhibitors (CNIs) have been essential components of immunosuppression. However, CNIs have been associated with an increased risk of nephrotoxicity, diabetes, hypertension, neurotoxicity, and de novo and recurrent malignancy [1]. Therefore, significant research efforts have been devoted to developing CNI-sparing and CNI-free approaches to immunosuppression. Currently, recommended strategies combine low-exposure CNIs with other agents, including mycophenolic acid derivatives and mammalian target of rapamycin (mTOR) inhibitors [2].

Everolimus, the first mTOR inhibitor (mTORi) to be licensed to prevent graft rejection following liver transplantation, has shown comparable efficacy in combination with reduced-exposure TAC and a nephroprotective effect versus standard-exposure CNI-based immunosuppression in clinical trials in liver transplant (LT) recipients [3, 4]. In 2014, following the introduction of everolimus, a group of transplant physicians, along with representatives of Italian LT centers, convened as the Italian Liver Transplant Working Group to launch the Everolimus & Liver: Expert Consensus TRAnsposition (ELECTRA) project [2]. The group produced evidence- and consensus-based recommendations to facilitate the integration of everolimus into the existing immunosuppressive regimens [2]. In 2020, the Italian Liver Transplant Working Group published, with the endorsement of the Italian Society for Organ and Tissue Transplantation (SITO), practice-oriented algorithms for immunosuppressive therapy in adult LT recipients [5]. The algorithms were directed to the following groups of transplant recipients: standard (low risk); critical (high risk); with an uncommon indication for LT; with hepatocellular carcinoma (HCC); and with de novo malignancy following LT. The Working Group pointed out the need for a regular update of the recommendations (ideally every 2 years), due to the rapid evolution of the LT field.

This article presents an update of the 2020 recommendations [2, 5]. It presents a series of evidence- and consensus-based statements addressing topics identified by the Working Group as new issues concerning immunosuppressive therapies and discusses the supporting literature. The article also presents updated treatment algorithms.

## Methods

The Italian Liver Transplant Working Group used the strategy described in previous publications [2, 5]. Briefly, the ELECTRA scientific board (U.C., L.D.C, P.D.S., M.D.G., S.F, F.L., G.T., and R.V.) identified issues of immunosuppressive therapy that needed to be updated, based on a review of the recent literature and on the specialists’ personal experiences. The following topics were identified: (i) new oncologic indications for LT; (ii) management of chronic LT rejection; (iii) combined liver–kidney transplantation; (iv) transplantation with an organ donated after circulatory death (DCD); (v) transplantation in the presence of frailty and sarcopenia; and (vi) ABO blood group incompatibility (ABO-I) between donor and recipient. The scientific board and representatives of Italian LT centers developed a series of statements addressing the identified issues. On May 24, 2022, in a plenary meeting attended by the scientific board and a panel of 20 experts in LT, the statements were voted in a modified Delphi process [2]. To merge new developments on the matter, the scientific board re-evaluated the statements in early 2024*.* The quality of the evidence supporting each statement and the statement's strength were evaluated using the Grading of Recommendations Assessment, Development, and Evaluation (GRADE) system [6]. Updated algorithms for immunosuppressive therapy were developed by the scientific board and finalized based on the consensus among all Italian Liver Transplant Working Group members.

## Oncologic indications for LT

Transplant oncology is currently attracting considerable interest as a treatment option for primary and secondary liver malignancies. In Europe, the proportion of oncologic indications for LT has significantly increased over the past decade [7]. Besides HCC, which is the second most common indication for LT in Europe after cirrhosis [7], other oncologic indications for LT are intrahepatic cholangiocarcinoma [8], perihilar cholangiocarcinoma [9], liver metastases of several tumor types (including gastrointestinal stromal tumor [GIST] [10], neuroendocrine tumor [NET] [11], and colorectal cancer [12, 13]), and hepatoblastoma [14]. Consensus statements on oncologic indications for liver transplantation are shown in Table 1.

**Table 1:** Consensus statements on the oncologic indications for liver transplantation

Oncologic indication	Statement	Level of evidence	Strength of statement
HCC	1.1 Low-exposure CNI with an mTORi is recommended for patients undergoing LT for HCC	Moderate	Strong
	1.2 mTORi-based immunosuppressive regimens should be considered for patients undergoing LT for high-risk HCC (AFP > 400 mg/ml; Milan-Out; ± microvascular invasion; G2–G3)	Moderate	Strong
	1.3 Steroid-free regimens do not significantly improve HCC recurrence rates	Moderate	Weak
	1.4 ACR episodes should be prevented as the steroid boluses required for their treatment significantly increase the risk of HCC recurrence	Low	Weak
pCCA/iCCA	1.5 mTORis are indicated due to antiproliferative properties; when systemic antitumor therapies are required, caution is needed to avoid potential cumulative AEs	Low	Weak
GIST-LM	1.6 Treatments acting on the PI3K/Akt/mTOR pathway have proven efficacious; use of conventional mTORi-based immunosuppressive therapy has a rationale in this setting	Very low	Strong
NET-LM	1.7 mTORi-based immunosuppressive therapy is potentially beneficial	Very low	Strong
CRC-LM	1.8 No evidence to support the use of particular immunosuppressive therapy for patients undergoing LT for CRC-LM. Further studies are needed in this regard	Very low	Strong

*ACR* acute cellular rejection, *AFP* alpha-fetoprotein, *Akt* protein kinase B, *CNI* calcineurin inhibitor, *CRC* colorectal cancer, *GIST* gastrointestinal stromal tumor, *HCC* hepatocellular carcinoma, *iCCA* intrahepatic chonalgiocarcinoma, *LM* liver metastasis, *LT* liver transplant, *mTOR* mammalian target of rapamycin, *mTORi* mTOR inhibitor, *NET* neuroendocrine tumor, *pCCA* perihilar cholangiocarcinoma, *PI3K* phosphoinositide 3-kinase

Immunosuppressive therapy should prevent graft rejection while minimizing the risk of disease recurrence in patients undergoing LT due to liver cancer. mTORis, which combine immunosuppressive activity and antiproliferative properties, should be able to fulfill this double requirement in oncology patients [15].

### HCC

Published evidence on strategies for preventing HCC recurrence in LT patients is lacking [3]. HCC recurrence mainly occurs during the first 2 years following LT at an estimated rate of 8–20% [3]. In the absence of guiding evidence, immunosuppression should be selected based on a personalized approach that takes into account several factors, including patient conditions, the Model for End-stage Liver Disease (MELD) score, tumor burden, response after locoregional treatment according to the modified Response Evaluation Criteria in Solid Tumors (mRECIST), alpha-fetoprotein (AFP) levels, and the time spent on the waiting list before LT [16, 17].

mTORis have been shown to lower HCC recurrence in LT patients with HCC [4, 18, 19]. For example, in the phase 3 SiLVER trial, the incorporation of sirolimus was associated with longer 3-year recurrence-free survival and overall survival (OS) following LT, especially for patients within the Milan criteria [18]. The impact of corticosteroids on HCC recurrence is not clearly defined (so is not recommended). No statistically significant differences in disease-free survival and OS have been reported in studies comparing steroid-free versus steroid-containing immunosuppressive regimens [20]. However, treatment of acute rejection episodes with steroids might increase the risk of post-transplant HCC recurrence**.** A recent study reported an 18-fold increase in the incidence of HCC recurrence in LT recipients treated with corticosteroid boluses for acute cellular rejection (ACR) compared with patients who did not receive corticosteroids [21].

### Intrahepatic and perihilar cholangiocarcinoma

LT following a specific protocol of neoadjuvant chemo-radiotherapy can be considered for selected patients with unresectable intrahepatic and perihilar cholangiocarcinoma [8, 9, 22–24]. In centers with extensive experience in this type of intervention, 5-year progression-free survival (PFS) and OS rates of 63% and 53%, respectively, have been reported [25, 26]. Although there is currently no general consensus on the preferred immunosuppressive treatment following LT due to cholangiocarcinoma, mTORis appear to be an option due to their antiproliferative properties and the positive impact on survival reported in preclinical [27, 28] and clinical studies [8, 29, 30].

### Liver metastases

GIST-related liver metastases are a rare indication for LT; transplantation can be considered for inoperable and imatinib-refractory metastases, although published evidence is very limited [10, 31]. mTORis have shown therapeutic potential for patients who underwent LT for GISTs [32].

LT eligibility for patients with neuroendocrine liver metastases is based on stringent selection criteria that take into account markers (^68^Ga-DOTATATE, Ki67), histologic findings, site of the primary tumor, and response to therapies [33, 34]. In the RADIANT trials, the use of mTORis was associated with a significant improvement in PFS and a greater proportion of patients with stable disease versus placebo; in the RADIANT-4 trial, mTORi use was associated with a 52% reduction in mortality among patients with advanced NET affecting the lungs and gastrointestinal system [35, 36].

The development of colorectal cancer liver metastases has a detrimental impact on patient survival [37]. In highly selected patients, and within specific protocols (such as those used in the COLT and MELODIC trials), LT may be an option [12, 13, 38]. Evidence supporting a specific immunosuppressive regimen in these patients is currently lacking [39].

## Management of chronic liver transplant rejection

The estimated incidence of chronic rejection is < 5%, but the actual incidence may be greater [40]. Consensus statements on this topic are shown in Table 2.

**Table 2:** Consensus statements on the management of chronic liver transplant rejection

	Statement	Level of evidence	Strength of statement
Diagnosis of chronic rejection	2.1 Diagnosis of chronic rejection should always be confirmed by biopsy	Moderate	Strong
	2.2 The minimum histologic criteria that define cell-mediated chronic rejection are (all criteria must be present): Disappearance of the bile duct in > 50% of the portal space Obliterative arterial disease	Moderate	Strong
	2.3 The criteria that define antibody-mediated chronic rejection are (all criteria must be present): Histopathologic pattern characterized by: Mononuclear cell infiltrate of any degree in the portal and/or perivenular space, not otherwise explainable, with necroinflammatory activity at the interface and/or in the perivenular space Portal/periportal, sinusoidal and/or perivenular fibrosis of at least a moderate degree Recent detection of serum DSAs (within 3 months from biopsy) At least focal positivity for C4d component (> 10% of the microvascular endothelium of the portal space) Exclusion of other possible causes	Moderate	Strong
	2.4 The non-invasive biomarkers for the diagnosis of antibody-mediated chronic rejection are de novo DSAs against class II-HLA antibodies	Low	Conditional
Role of DSAs in chronic rejection	2.5 Risk factors for developing de novo DSAs include: Low MELD score (< 15) at transplantation Previous transplant Young age (< 60 years) Low immunosuppressant level	Moderate	Strong
	2.6 The presence of de novo DSAs correlates with the occurrence of ACR, antibody-mediated chronic rejection, and early biliary complications after transplantation	Low	Conditional
	2.7 After LT, the long-term persistence of DSAs correlates with the development of fibrosis	Low	Conditional
	2.8 Sequential monitoring of fibrosis (by biopsy or elastography) is recommended for implementing changes in immunosuppressive therapy and/or evaluating the timing of retransplantation	Very low	Weak
Immunosuppressive therapy	2.9 In patients diagnosed with cell-mediated chronic rejection, a gradual increase in immunosuppressive therapy can be considered; large fluctuations in serum levels of immunosuppressant should be avoided	Low	Strong
	2.10 Patients with cell-mediated chronic rejection treated with CsA monotherapy can be switched to tacrolimus	Moderate	Strong
	2.11 mTORi treatment has resulted in the control of cell-mediated chronic rejection in up to 50% of non-responders	Low	Conditional
	2.12 MFI ≥ 5000 may have clinical relevance for the diagnosis of antibody-mediated chronic rejection. In case of MFI ≥ 5000, the CNI dose should be increased (if tolerated), or MFA should be added	Low	Conditional

*ACR* acute cellular rejection, *C4d* complement 4d, *CNI* calcineurin inhibitor, *CsA* cyclosporine A, *DSAs* donor-specific antibodies, *HLA* human leukocyte antigen, *LT* liver transplant, *MELD* model of end-stage liver disease, *MFI* mean fluorescence intensity, *MFA* mycophenolic acid, *mTOR* mammalian target of rapamycin, *mTORi* mTOR inhibitor

### Diagnosis of chronic rejection

Elevation of liver enzymes in mid/long-term LT recipients may indicate chronic rejection when other transplantation-related complications are excluded. Risk factors for chronic rejection with a preeminent cell-mediated component include: history of ACR; underlying autoimmune disease; poor adherence to immunosuppressive therapy; treatment with cyclosporine; retransplantation due to graft rejection; advanced age of the donor (> 40 years); and gender mismatch. For chronic rejection with a preeminent antibody-mediated component, the risk factors to be considered are post-transplantation development of donor-specific antibodies (DSAs); low blood levels of CNIs; low MELD score (< 15) at LT; young age of recipient; and retransplantation. The diagnosis of chronic rejection should always be confirmed by liver graft biopsy [41, 42].

### Role of DSAs in chronic rejection

DSAs, extensively studied in kidney transplantation, are attracting interest in LT [43, 44]. Most pre-existing DSAs are class I anti-human leukocyte antigen (HLA) antibodies [43]. Of these, only 5% persist following LT and do not seem to be involved in the development of chronic rejection. Instead, de novo DSAs, which are mostly directed against class II-HLA antibodies, should always be investigated when antibody-mediated chronic rejection is suspected. These DSAs are rarely produced during the first 6 months following LT, while their incidence can reach 40% beyond 15 years. The presence of de novo DSAs correlates with the development of biliary complications and the development of antibody-mediated chronic rejection, and their persistence predicts the development of graft fibrosis [43]. In mid/long-term recipients, altered liver function tests (i.e., a twofold liver enzyme activity increase), in the absence of other transplantation-related complications, should always prompt the monitoring of de novo DSAs [43, 45].

### Retransplantation due to chronic rejection

Before the introduction of CNIs, chronic rejection was the leading indication for liver retransplantation. Retransplantation is the only option in cases of end-stage liver disease due to severe chronic rejection [46]. Compared with primary LT, retransplantation has significantly worse outcomes in terms of risk of organ loss at 1 year and patient survival [7, 47]. Predictive tools are required to improve the selection of patients eligible for liver retransplantation [48].

## Combined liver–kidney transplantation

A review of the literature failed to find novel immunosuppressive strategies in patients requiring combined liver–kidney transplantation. Therefore, no significant changes were made to the algorithm proposed in 2020 for these patients [5]. According to the updated algorithms presented here, patients with combined liver–kidney transplantation are categorized as “patients with specific etiology” (described in detail below) and are treated as standard or critical patients, based on a multidisciplinary decision.

## Immunosuppression for DCD-liver recipients

According to published evidence and in our experience, most problems related to the use of DCD-organs arise from ischemia–reperfusion injury [49]. Strategies for preserving DCD-organs based on normothermic regional perfusion, mandatory in Italy because of our 20-min no-touch period, followed by re-conditioning ex situ in hypo- or normothermia, have been developed and play a crucial role in the recovery of organs that would otherwise be discarded [50]. Uncontrolled DCDs here remain anecdotal [51]. A consensus paper by the International Liver Transplantation Society suggests the use of a controlled DCD (cDCD)-organ in patients with MELD ≤ 25 and recommends avoiding the use of grafts with > 30% steatosis, in the absence of a device for perfusion [49].

Recipients of DCD-livers should be categorized as critical patients and receive the immunosuppressive regimens suggested for this group (described in detail below), using induction to mitigate early graft failure in cDCD LT and to defer the start of CNIs and preserve renal function. However, DCD-organs are preferentially assigned to low-risk patients, who can be managed according to the recommendations for standard patients and for whom induction is indicated only in case of intraoperative complications. Evidence suggests that induction therapy with anti-thymocyte globulin can improve the outcomes of LT with a DCD-organ [52]. Until the physiologic mechanism underlying biliary complications is well defined, any important role for immunomodulation remains speculative and deserves further investigation [53].

Consensus statements on immunosuppressive therapies in DCD-liver recipients are shown in Table 3.

**Table 3:** Consensus statements on immunosuppression for liver transplantation with an organ donated after circulatory death

	Statement	Level of evidence	Strength of statement
Rejection risk and immunosuppressive regimens	3.1 Inflammasome-related changes in the DCD-graft (ischemia–reperfusion injury) may induce specific interactions with the host immune system, with a possible increase in the risk of rejection	Low	Weak
	3.2 Induction therapy with anti-thymocyte globulin can improve outcomes	Low	Conditional
Specific strategies	3.3 Hypothermic oxygenated machine perfusion may reduce the risk of biliary non-anastomotic strictures	High	Conditional
	3.4 Normothermic machine perfusion has been shown to limit the extent of ischemia–reperfusion injury and proinflammatory responses; this immunomodulatory effect may reduce the occurrence of organ rejection, thus allowing a decrease of immunosuppressant dosage	High	Conditional
	3.5 Normothermic regional perfusion (also used in sequence with machine perfusion) may reduce biliary complications and DCD-organ loss	High	Conditional
	3.6 In the absence of machine perfusion, the use of DCD-grafts with > 30% macrovesicular steatosis should be avoided	Moderate	Conditional
Organ selection	3.7 Routine use of cDCD-livers is recommended for transplant candidates with a laboratory MELD score ≤ 25	High	Strong
	3.8 Allocation of cDCD-organs to transplant candidates with advanced liver disease and a MELD score > 25 should be carefully evaluated	Moderate	Conditional

*DCD* donated after circulatory death, *MELD* model of end-stage liver disease

## Transplantation in the presence of frailty and sarcopenia

The average age of LT recipients is steadily increasing. In the 1980s, patients aged > 60 years represented < 5% of the LT population in Europe, while in 2015 they represented > 30% [7]. Older patients are often affected by frailty and sarcopenia. Frailty refers to a syndrome of reduced resistance to stressors caused by the cumulative impairment of multiple systems and resulting in vulnerability to adverse outcomes [54]. Several tools for measuring frailty are available [54–56]. Sarcopenia is defined by the European Working Group on Sarcopenia as “progressive and generalized disorder of skeletal muscles, associated with a greater likelihood of adverse events, including falls, fractures, disability, and mortality” [57]. Frailty and sarcopenia are not absolute contraindications for LT. Sarcopenia and frailty are, however, independent predictive factors of mortality during the period on the waiting list and following LT [58–61]. Furthermore, in the post-transplantation period, frailty and sarcopenia have been associated with increased intubation time (> 24 h), need for tracheostomy, > 5-day stay in the intensive care unit, and prolonged hospital stay (> 20 days), mostly due to cardiovascular and neurologic complications [62, 63]. Based on these considerations, it is crucial to be aware of sarcopenia and frailty when evaluating older candidates for LT; if these conditions are present, their severity should be assessed. Consensus statements on LT in the presence of frailty and sarcopenia are shown in Table 4.

**Table 4:** Consensus statements on immunosuppression in frail liver transplant recipients with sarcopenia

	Statement	Level of evidence	Strength of statement
Impact of sarcopenia on LT	4.1 The presence of severe sarcopenia should not be considered an absolute contraindication to LT	Moderate	Strong
	4.2 Objective evaluation of sarcopenia before LT can be useful for predicting post-transplantation clinical outcomes	Moderate	Weak
	4.3 Waitlisted patients with MELD-Na scores < 20 with sarcopenia may have priority over waitlisted patients with an identical MELD score and no sarcopenia	Moderate	Weak
	4.4 Waitlisted patients with MELD-Na scores > 35 with sarcopenia and/or additional comorbidities may be considered ineligible for LT, given the expected high post-transplantation mortality	Low	Weak
Strategies for managing sarcopenia	4.5 The PONS score should be calculated to evaluate nutritional deficiencies	Moderate	Weak
	4.6 Most patients with sarcopenia before LT will have persistent sarcopenia after the intervention. Many patients with cirrhosis and without sarcopenia before LT, will develop new-onset sarcopenia subsequently. Therefore, sarcopenia monitoring is also recommended after LT	Moderate	Strong
	4.7 In the presence of malnutrition, appropriate nutritional interventions should be initiated. If oral nutrition fails to meet patient needs, enteral nutrition is preferred over the parenteral approach. If parenteral nutrition is required, administer for 7–14 days and supplement with oral or enteral nutrition, if possible	Moderate	Strong
	4.8 The combination of nutrition and exercise is the best strategy for reducing pre- and post-LT sarcopenia	Moderate	Strong
Immunosuppression and sarcopenia	4.9 Infections are the leading cause of death in sarcopenic patients after LT. Close monitoring of infections and prompt treatment are recommended	Moderate	Strong
	4.10 In the case of sarcopenic LT recipients, older donors should be avoided	Moderate	Strong
	4.11 Patients with sarcopenia undergoing LT do not require lower immunosuppressant target blood level, as they have an adjusted 3.3-fold increased risk of ACR within 3 months from LT, compared with patients without sarcopenia	Low	Weak
	4.12 mTORis alter the metabolism of skeletal muscle proteins, thereby contributing to the loss of muscle mass. Therefore, mTORis should not be used immediately after LT	Low	Weak
	4.13 Leucine supplementation may have beneficial effects on body composition, due to its regulatory activity on mTOR-mediated signal transduction	Low	Weak

*ACR* acute cellular rejection, *LT* liver transplant, *MELD* model of end-stage liver disease, *mTOR* mammalian target of rapamycin, *mTORi* mTOR inhibitor, *PONS* perioperative nutrition screen

### Immunosuppression and sarcopenia

Regardless of its severity, frailty seems to increase graft rejection risk by more than threefold in the first 3 months following LT because of the dysregulation of the immune system [64]. In frail patients with sarcopenia, immunosuppressive treatment should be determined by considering the reduction in muscle mass and the occurrence of complications typically associated with sarcopenia [63]. A program of nutritional advice and physical exercise could be set up while the patient is on the LT waiting list [64]. To this purpose, perioperative nutritional screening (PONS) is a rapid and effective tool for detecting nutritional deficiency [65].

With regard to immunosuppressive treatment in patients with sarcopenia, mTORis such as sirolimus and everolimus (which mainly act by inhibiting the target of rapamycin complex TORC1) can alter muscle metabolism and contribute to the reduction of muscular mass [15, 66]. Findings from preclinical studies have led to recommendations against the use of mTORis in the early post-transplantation phase; however, further clinical evidence is needed to clarify this point [67].

With regard to immunosuppressive treatment in patients with sarcopenia, muscle wasting leads to reduced drug distribution volume and higher blood concentrations. It is recommended to perform drug exposure testing more frequently in these patients to avoid overexposure to immunosuppressants [2].

## Management of patients with ABO blood group incompatibility with donors

According to the 2018 report of the European Liver Transplant Registry, 93% of LTs are isogroup and 6.5% are ABO compatible; ABO-I transplants account for 3% of LTs that are performed due to emergencies [7]. In both elective and emergency conditions, isogroup LTs have a significantly better 5-year survival compared with ABO compatible or ABO-I LTs (66% vs 62% and 57%, respectively, *p* < 0.0001; and 56% vs 53% and 28%, respectively, *p* = 0.001) [7].

Published evidence on immunosuppression in transplanted ABO-I patients is lacking, and practical experience in transplant centers in Italy is limited. For this reason, these patients were not included in the 2020 version of the recommendations by the Italian Liver Transplant Working Group [5]. However, the emergency status of ABO-I patients requiring LT and the paucity of supporting data make the need for a standard and shared protocol among Italian LT centers even more important. As the critical condition of these patients is due mainly to their immunologic risk, recommendations for post-transplantation management address both immunosuppression and immunomodulation.

## Algorithms for immunosuppressive therapy

**Fig. 1:**
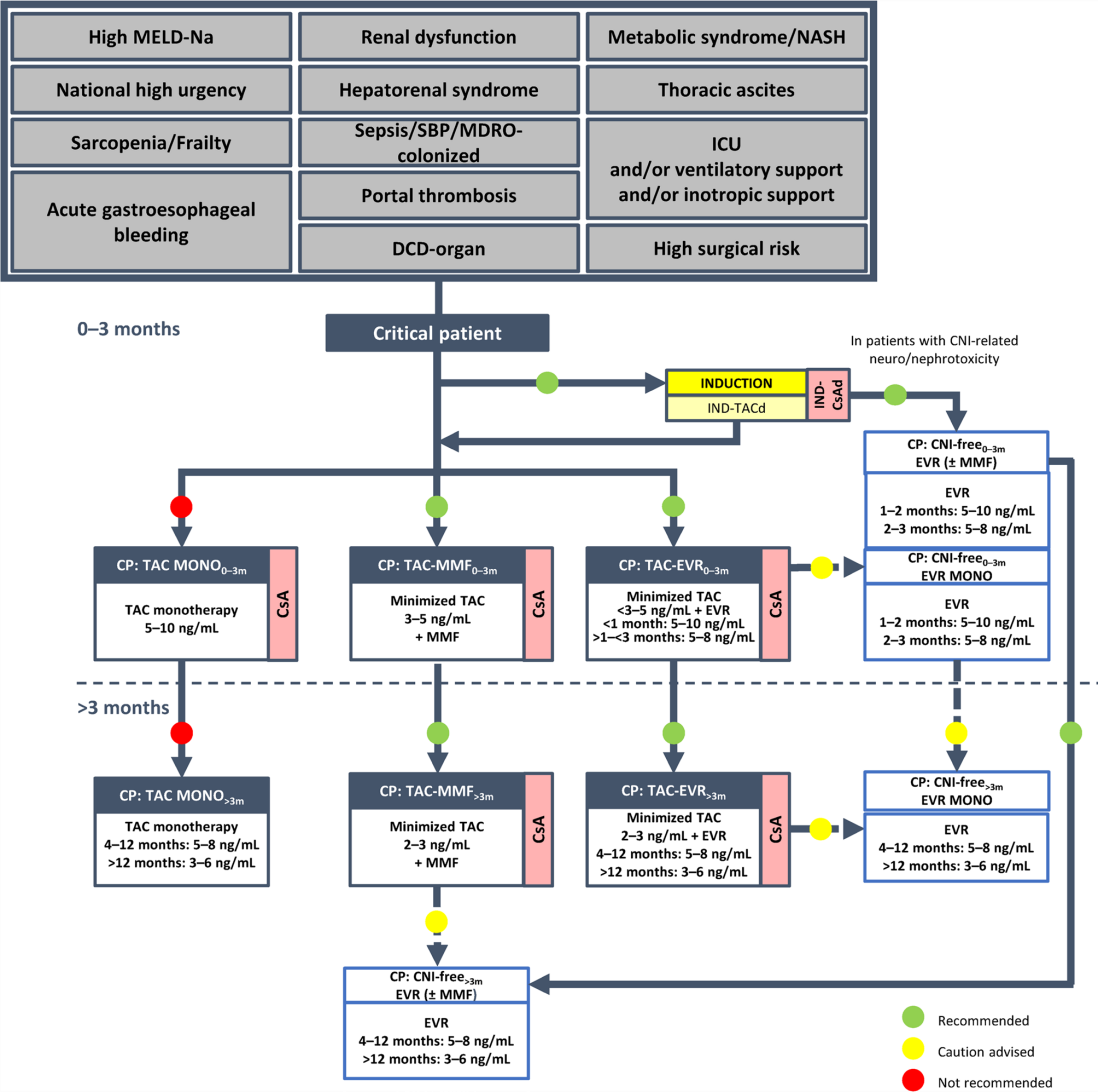
Algorithm for immunosuppressive therapy in critical patients undergoing liver transplantation (as defined in Supplementary methods). Induction is indicated to allow delayed calcineurin (CNI) introduction, early CNI minimization, and a steroid-free approach. Critical patients with an infection contracted after the transplantation should be considered for reduction/discontinuation of immunosuppressive therapy. The indicated target blood levels of immunosuppressants are not binding. In these patients, induction therapy with basiliximab to delay the introduction of CNIs by a few days is recommended. CNI reduction with the introduction of mycophenolate mofetil or everolimus is also recommended, while CNI monotherapy should be avoided. In critical patients with CNI-related neurotoxicity and/or nephrotoxicity a CNI-free regimen with everolimus in monotherapy or combined with mycophenolate mofetil is recommended, following induction therapy. A CNI-free regimen based on everolimus with or without mycophenolate mofetil may eventually be considered, with some caution, for patients receiving other regimens within this protocol, especially at > 3 months post-transplantation. *BMI* body mass index, *CsA* cyclosporine, *CP* critical patient, *d* delayed, *DCD* donated after circulatory death, *eGFR* estimated glomerular filtration rate, *EVR* everolimus, *ICU* intensive care unit, *IND* induction, *MDRO* multidrug-resistant organism, *MELD-Na* model for end-stage liver disease-sodium, *MMF* mycophenolate mofetil, *MONO* monotherapy, *NASH* non-alcoholic steatohepatitis, *NCEP-ATP III* National Cholesterol Education Program: Adult Treatment Panel III, *NKDOQI* National Kidney Disease Outcomes Quality Initiative, *SBP* spontaneous bacterial peritonitis, *SPPB* Short Physical Performance Battery, *TAC* tacrolimus

**Fig. 2:**
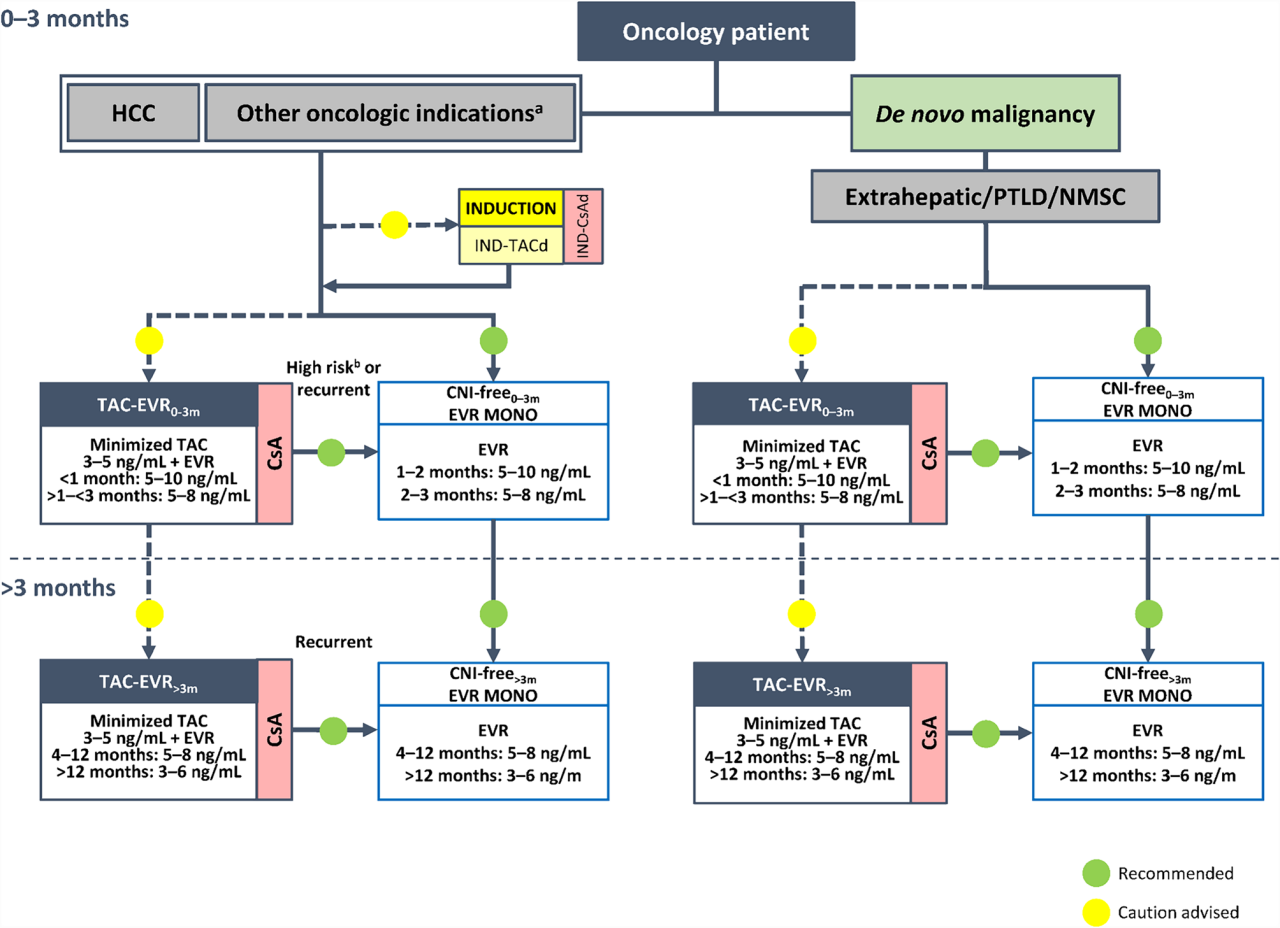
Algorithm for immunosuppressive therapy in oncology patients undergoing liver transplantation. The indicated target blood levels of immunosuppressants are not binding. A protocol of CNI reduction with everolimus is recommended. While CNI-containing and CNI-free regimens were both recommended in the 2020 version of the algorithm for patients with HCC, [5], a CNI-free regimen with everolimus monotherapy is the preferred option in the updated algorithm, especially in patients with high-risk or recurrent oncologic disease, owing to the antiproliferative properties of mTOR inhibitors. ^a^Intrahepatic cholangiocarcinoma, perihilar cholangiocarcinoma, hepatoblastoma, and liver metastases of NET, GIST, or colorectal cancer. ^b^For patients with HCC or NET. *CNI* calcineurin inhibitor, *CsA* cyclosporine, *d* delayed, *EVR* everolimus, *GIST*, gastrointestinal stromal tumor, *HCC* hepatocellular carcinoma, *IND* induction, *mTOR* mammalian target of rapamycin, *MONO* monotherapy, *NET* neuroendocrine tumor, *NMSC* non-melanoma skin cancer, *PTLD* post-transplant lymphoproliferative disorder, *TAC* tacrolimus

**Fig. 3:**
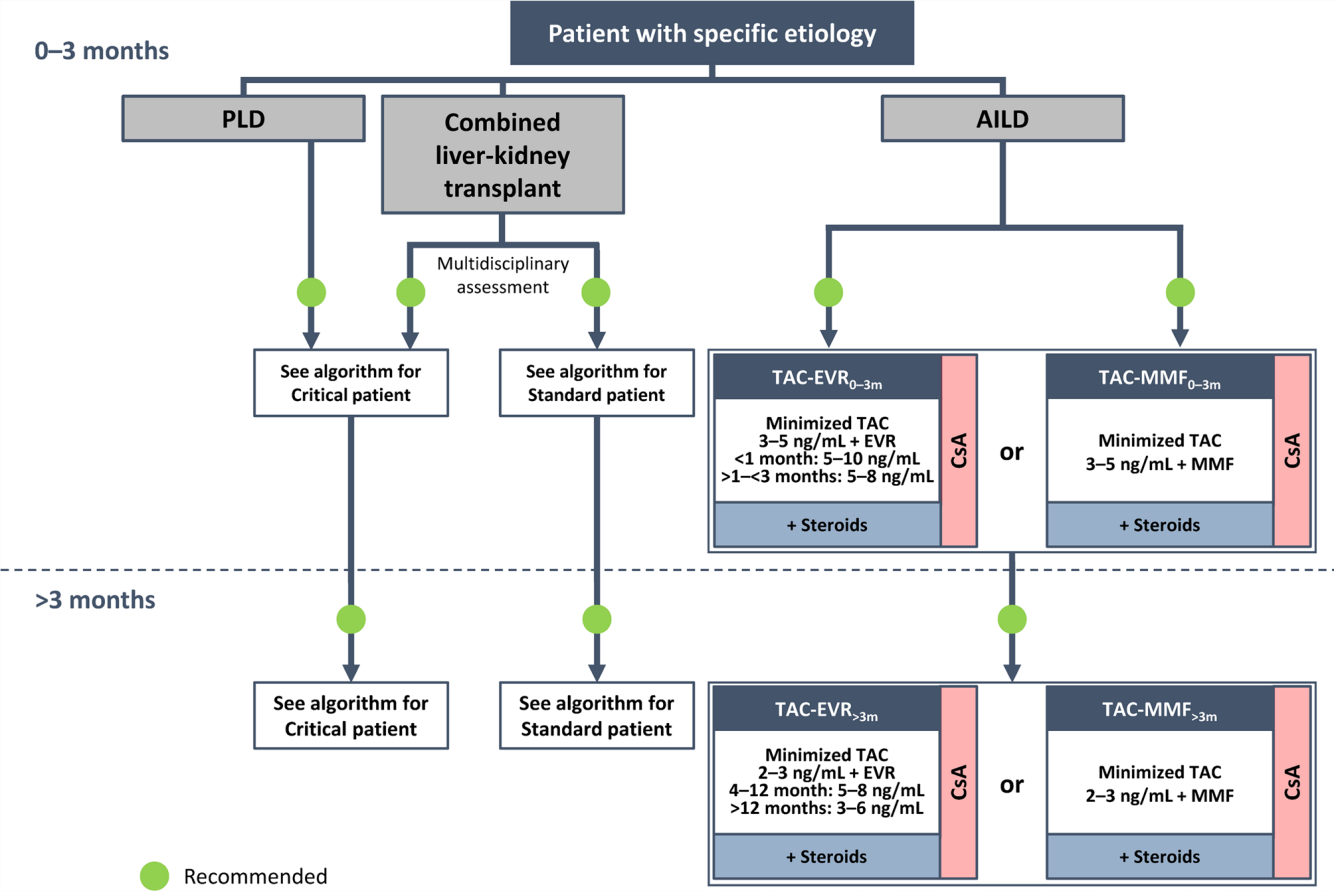
Algorithm for immunosuppressive therapy in patients with specific etiology undergoing liver transplantation. The indicated target blood levels of immunosuppressants are not binding. Patients requiring liver–kidney transplantation should be treated according to the protocol for critical patients or standard patients, depending on the patient clinical status and after evaluation by a multidisciplinary team [5]. The immunosuppressive protocol for patients with AILD is the same as that recommended for standard patients with the addition of corticosteroids at a dose that should be adjusted based on efficacy and reported adverse events at 0–3 months and > 3 months post-transplantation. *AILD* autoimmune liver disease, *CNI* calcineurin inhibitor, *CsA* cyclosporine, *EVR* everolimus, *MMF* mycophenolate mofetil, *PLD* polycystic liver disease, *TAC* tacrolimus

**Fig. 4:**
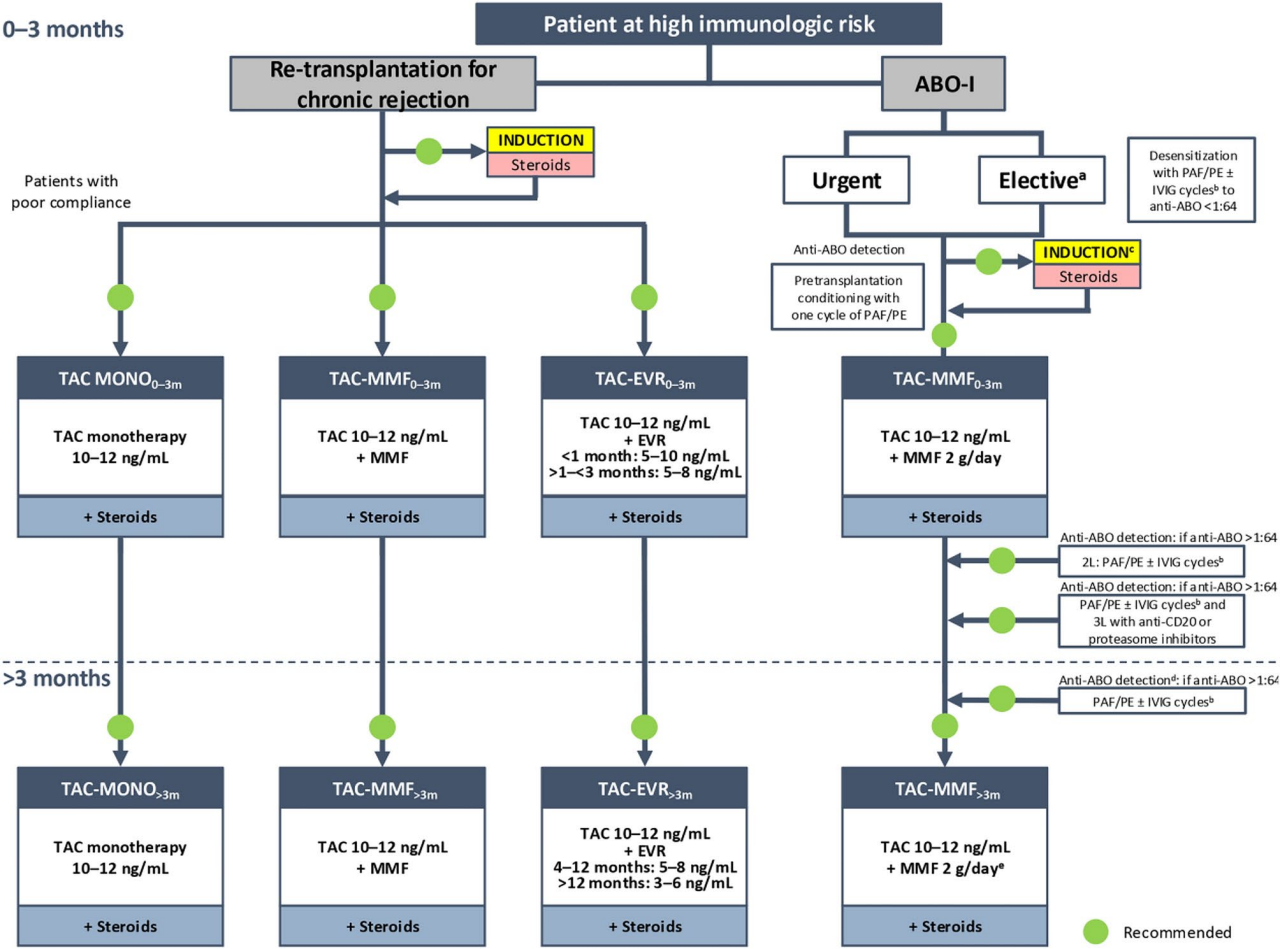
Algorithm for immunosuppressive therapy in patients at high immunologic risk undergoing liver transplantation. The indicated target blood levels of immunosuppressants are not binding. Patients with chronic graft rejection should first receive induction therapy (basiliximab) plus corticosteroids followed by tacrolimus in monotherapy plus corticosteroids for patients with compliance problems, or CNI-reducing regimens with tacrolimus and mycophenolate mofetil or everolimus, plus corticosteroids in both cases. In ABO-I patients, transplant surgery can be urgent or elective. Candidates for elective surgery should be desensitized with cycles of plasmapheresis/plasma exchange with or without immunoglobulins to reach an anti-ABO titer < 1:64. Patients needing an emergency intervention should be conditioned preoperatively with one cycle of plasmapheresis/plasma exchange. Following conditioning, the immunosuppression protocols for emergency and elective liver transplants are similar and involve induction therapy plus corticosteroids followed by tacrolimus–mycophenolate mofetil with corticosteroids. Monitoring of anti-ABO titer is recommended; if the titer is > 1:64, second-line immunomodulatory treatment involves cycles of plasmapheresis/plasma exchange with or without immunoglobulins. If the ABO titer continues to be > 1:64, third-line treatment is recommended with use of anti-CD20 agents or proteosome inhibitors. At > 3 months post-transplantation, the recommended regimens remain the same as during the 3 months following the intervention, with a decrease in the tacrolimus dose. ^a^Organ from a living donor. ^b^Frequency and duration of PAF/PE depend on anti-A and anti-B titers. ^c^The use of polyclonal antibodies (ALG, ALT) requires monitoring and caution due to the high risk of infections and related complications. ^d^Repeat weekly during the first month. ^e^Recommended dose (may vary depending on individual center protocols). Adjustments of immunosuppressive therapy should be made based on patient clinical characteristics. *2L* second line, *3L* third line, *ABO-I* ABO incompatible, *ALG* antilymphocyte globulin*, ALT* alanine aminotransferase, *CNI* calcineurin inhibitor, *EVR* everolimus, *IVIG* intravenous immunoglobulin, *MMF* mycophenolate mofetil, *MONO* monotherapy, *PAF* plasmapheresis, *PE* plasma exchange, *TAC* tacrolimus

Algorithms were finalized by consensus for immunosuppressive therapies in the following categories of adult LT recipients: standard patients (Supplementary Fig. 1); critical patients (Fig. 1); oncology patients (Fig. 2); patients with specific etiology (Fig. 3); and patients at high immunologic risk (Fig. 4). With the exception of patients with autoimmune liver disease (AILD; Fig. 3) and at high immunologic risk (Fig. 4), a steroid-free approach is generally recommended.

### Standard patients

This category includes lower-risk patients with a MELD score < 25 and without an autoimmune disease, HCC, renal dysfunction, or a history of renal dysfunction, or any of the conditions affecting patients included in the other categories considered. The updated algorithm (Supplementary Fig. 1) also includes recipients of a DCD-organ, who are generally low-risk patients according to the recommended organ allocation criteria. No other changes have been made to the 2020 version of the recommendations [5].

### Critical patients

Patients with a MELD-sodium (Na) score > 29 or ≥ 25 to < 29 with concurrent renal dysfunction or chronic encephalopathy, or with one or more of the conditions listed at the top of Fig. 1, are considered critically ill patients, requiring particular care in the selection of the IT. Overall, the recommended immunosuppressive protocol for critical patients is similar to that of the 2020 version of the recommendations [5].

### Oncology patients

This category includes patients with HCC and with other oncologic indications for LT, including intrahepatic and perihilar cholangiocarcinoma, liver metastases from colorectal cancer, GIST, or NET, and hepatoblastoma (very rare in adults), as well as patients with de novo malignancies following LT. In the proposed algorithm (Fig. 2), oncologic indications for transplantation other than HCC have a more prominent position than in the 2020 version of the recommendations, which had assigned such patients to the category of patients “with specific etiology”, due to the rarity of the indication at that time [5]. This change has been prompted by the increasing evidence supporting the benefits and feasibility of LT in these settings. A similar strategy is recommended for oncology patients with a de novo malignancy after LT.

### Patients with specific etiology

This category constitutes patients with liver diseases that are uncommon indications for LT (Fig. 3), for which the experience in many transplantation centers may be still limited. These diseases include polycystic liver diseases (isolated polycystic liver disease and autosomal dominant polycystic kidney disease), conditions requiring the combined liver–kidney transplantation, and AILD. In line with the recommendations published in 2020, patients with polycystic disease should be treated according to the protocol recommended for critical patients (Fig. 1) [5].

### Patients at high immunologic risk

Patients at high immunologic risk were not considered in the 2020 version of the recommendations due to the lack of published evidence and expertise in most Italian transplant centers at that time [5]. In this version of the recommendations, two types of patients at increased immunologic risk have been included: patients with chronic graft rejection requiring liver retransplantation, and ABO-I patients (Fig. 4).

## Conclusion

This updated version of the 2020 consensus statements and algorithms address a range of variables to be considered in clinical practice to optimize the choice of immunosuppressive regimen in patients who have undergone LT. Clinicians are encouraged to refer to these recommendations to reduce heterogeneity that may be present in immunosuppressive treatment protocols that are used to treat ABO-compatible LT recipients at different transplant centers throughout Italy.

## Supplementary Information

Below is the link to the electronic supplementary material.

Supplementary file1 (DOCX 150 KB)

## Data Availability

Data sharing does not apply to this article as no datasets were generated or analyzed.
