# Correction: Immunosuppression in adult liver transplant recipients: a 2024 update from the Italian Liver Transplant Working Group

**DOI:** 10.1007/s12072-024-10766-3

**Published:** 2025-02-17

**Authors:** Tommaso Maria Manzia, Barbara Antonelli, Amedeo Carraro, Grazia Conte, Nicola Guglielmo, Andrea Lauterio, Laura Mameli, Umberto Cillo, Luciano De Carlis, Massimo Del Gaudio, Paolo De Simone, Stefano Fagiuoli, Francesco Lupo, Giuseppe Tisone, Riccardo Volpes, on behalf of the Italian Liver Transplant Working Group

**Affiliations:** 1 Department of Surgical Science, University of Rome Tor Vergata, Rome, Italy; 2 Fondazione IRCCS Ca’ Granda Ospedale Maggiore Policlinico, Milan, Italy; 3 Liver Transplant Unit, University Hospital Trust of Verona, Verona, Italy; 4 Clinica di Chirurgia Epatobiliare, Pancreatica e dei Trapianti, Azienda Ospedaliera Universitaria delle Marche, Ancona, Italy; 5 General Surgery and Liver Transplantation Unit, Azienda Ospedaliera San Camillo-Forlanini, Rome, Italy; 6 ASST Grande Ospedale Metropolitano Niguarda, University of Milano-Bicocca, Milan, Italy; 7 Azienda Ospedaliera G. Brotzu, Cagliari, Italy; 8 Hepatobiliary and Liver Transplant Unit, University Hospital of Padua, Padua, Italy; 9 Department of General Surgery and Transplantation, Niguarda Hospital, Milan, Italy; 10 School of Medicine, University of Milano-Bicocca, Milan, Italy; 11 Department of General Surgery and Transplantation, Policlinico S. Orsola-Malpighi, Bologna, Italy; 12 Hepatobiliary Surgery and Liver Transplantation Unit, University of Pisa Medical School Hospital, Pisa, Italy; 13 Gastroenterology, Department of Medicine, University of Milano-Bicocca and Gastroenterology Hepatology and Transplantation, Papa Giovanni XXIII Hospital, Piazza OMS, 124127 Bergamo, Italy; 14 Department of General Surgery, Azienda Ospedaliera Città Della Salute e Della Scienza, Turin, Italy; 15 Mediterranean Institute for Transplantation and Advanced Specialized Therapies (ISMETT/IRCCS), Palermo, Italy; 16 Fondazione Istituto G. Giglio di Cefalù, Palermo, Italy

**Correction: Hepatology International (2024) 18:1416–1430** 10.1007/s12072-024-10703-4

In this article Figs. 2, 3 and 4 were wrongly numbered; Fig. 2 should have been Fig. 1, Fig. 3 should have been Fig. 2 and Fig. 4 should have been Fig. 3. The Fig. 1 should be deleted and new Fig. 4 is provided in this corrections. Incorrect figures and correct figures are as shown below:

**Incorrect Figure 1** 
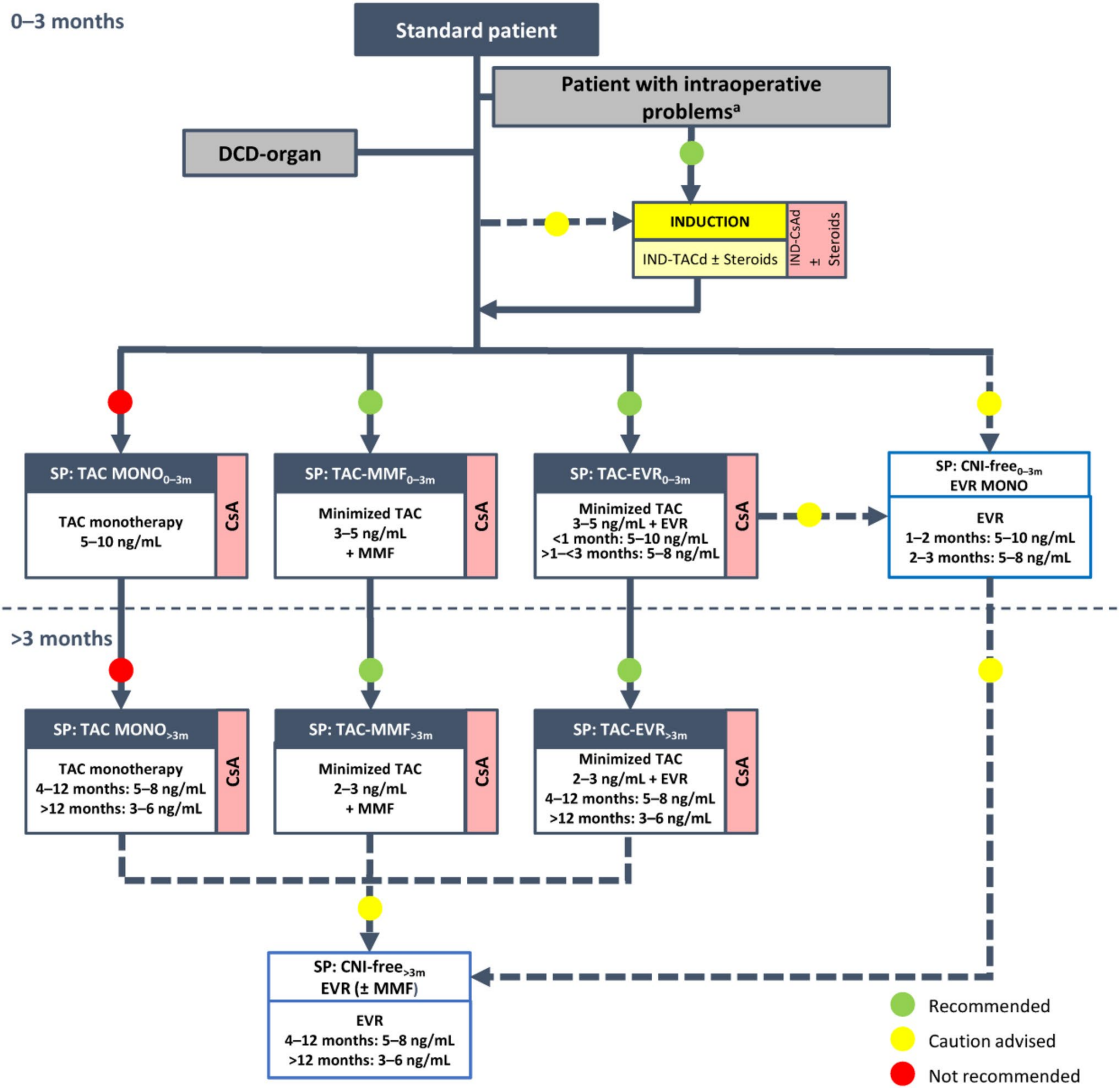


**Correct Figure 1**

**Fig. 1:**
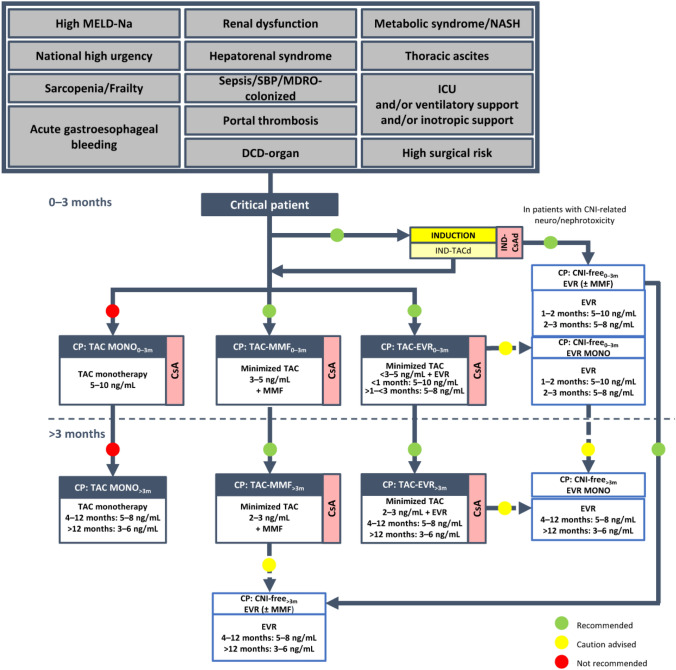
Algorithm for immunosuppressive therapy in critical patients undergoing liver transplantation (as defined in Supplementary methods). Induction is indicated to allow delayed calcineurin (CNI) introduction, early CNI minimization, and a steroid-free approach. Critical patients with an infection contracted after the transplantation should be considered for reduction/discontinuation of immunosuppressive therapy. The indicated target blood levels of immunosuppressants are not binding. In these patients, induction therapy with basiliximab to delay the introduction of CNIs by a few days is recommended. CNI reduction with the introduction of mycophenolate mofetil or everolimus is also recommended, while CNI monotherapy should be avoided. In critical patients with CNI-related neurotoxicity and/or nephrotoxicity a CNI-free regimen with everolimus in monotherapy or combined with mycophenolate mofetil is recommended, following induction therapy. A CNI-free regimen based on everolimus with or without mycophenolate mofetil may eventually be considered, with some caution, for patients receiving other regimens within this protocol, especially at > 3 months post-transplantation. *BMI* body mass index, *CsA* cyclosporine, *CP* critical patient, *d* delayed, *DCD* donated after circulatory death, *eGFR* estimated glomerular filtration rate, *EVR* everolimus, *ICU* intensive care unit, *IND* induction, *MDRO* multidrug-resistant organism, *MELD-Na* model for end-stage liver disease-sodium, *MMF* mycophenolate mofetil, *MONO* monotherapy, *NASH* non-alcoholic steatohepatitis, *NCEP-ATP III* National Cholesterol Education Program: Adult Treatment Panel III, *NKDOQI* National Kidney Disease Outcomes Quality Initiative, *SBP* spontaneous bacterial peritonitis, *SPPB* Short Physical Performance Battery, *TAC* tacrolimus

**Incorrect Figure 2** 
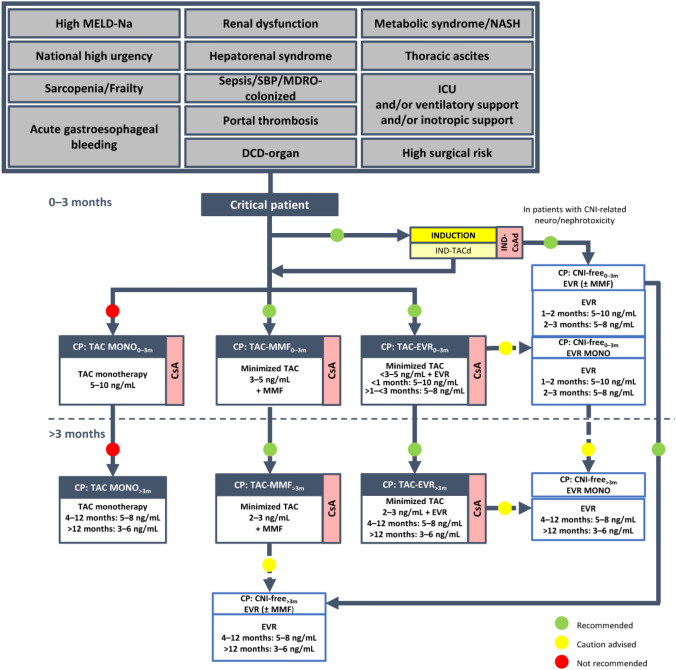


**Correct Figure 2**

**Fig. 2:**
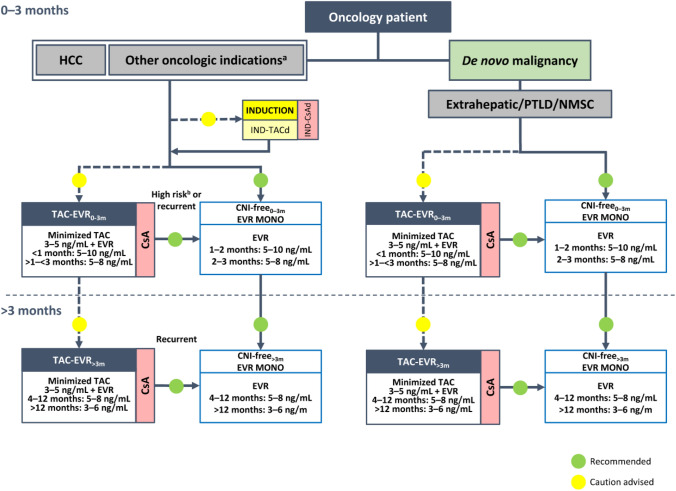
Algorithm for immunosuppressive therapy in oncology patients undergoing liver transplantation. The indicated target blood levels of immunosuppressants are not binding. A protocol of CNI reduction with everolimus is recommended. While CNI-containing and CNI-free regimens were both recommended in the 2020 version of the algorithm for patients with HCC, [5], a CNI-free regimen with everolimus monotherapy is the preferred option in the updated algorithm, especially in patients with high-risk or recurrent oncologic disease, owing to the antiproliferative properties of mTOR inhibitors. ^a^Intrahepatic cholangiocarcinoma, perihilar cholangiocarcinoma, hepatoblastoma, and liver metastases of NET, GIST, or colorectal cancer. ^b^For patients with HCC or NET. *CNI* calcineurin inhibitor, *CsA* cyclosporine, *d* delayed, *EVR* everolimus, *GIST*, gastrointestinal stromal tumor, *HCC* hepatocellular carcinoma, *IND* induction, *mTOR* mammalian target of rapamycin, *MONO* monotherapy, *NET* neuroendocrine tumor, *NMSC* non-melanoma skin cancer, *PTLD* post-transplant lymphoproliferative disorder, *TAC* tacrolimus

**Incorrect Figure 3** 
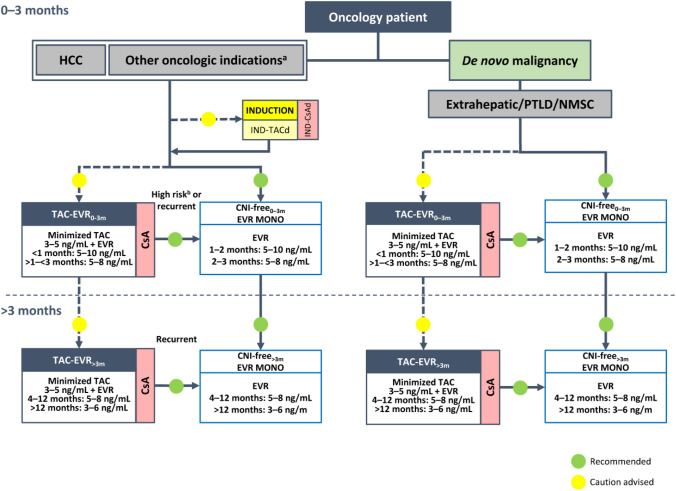


**Correct Figure 3**

**Fig. 3:**
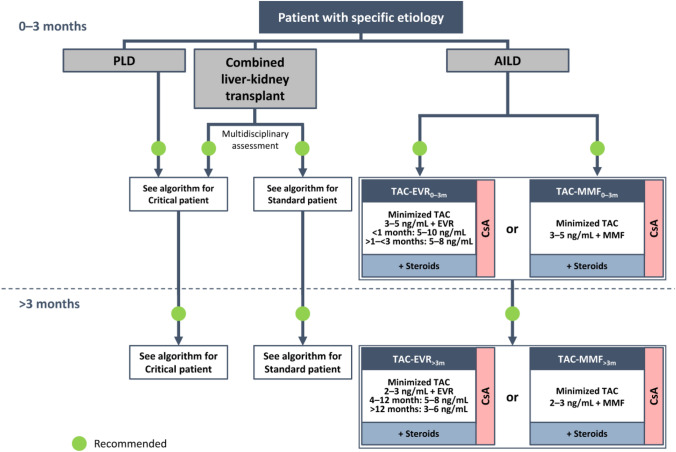
Algorithm for immunosuppressive therapy in patients with specific etiology undergoing liver transplantation. The indicated target blood levels of immunosuppressants are not binding. Patients requiring liver–kidney transplantation should be treated according to the protocol for critical patients or standard patients, depending on the patient clinical status and after evaluation by a multidisciplinary team [5]. The immunosuppressive protocol for patients with AILD is the same as that recommended for standard patients with the addition of corticosteroids at a dose that should be adjusted based on efficacy and reported adverse events at 0–3 months and > 3 months post-transplantation. *AILD* autoimmune liver disease, *CNI* calcineurin inhibitor, *CsA* cyclosporine, *EVR* everolimus, *MMF* mycophenolate mofetil, *PLD* polycystic liver disease, *TAC* tacrolimus

**Incorrect Figure 4** 
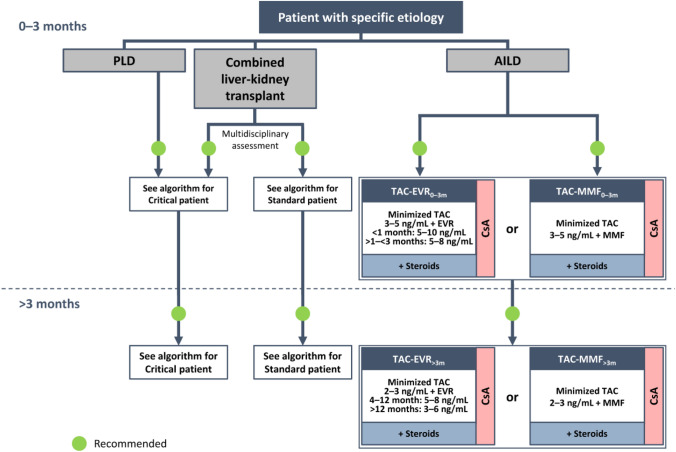


**Correct Figure 4**

**Fig. 4:**
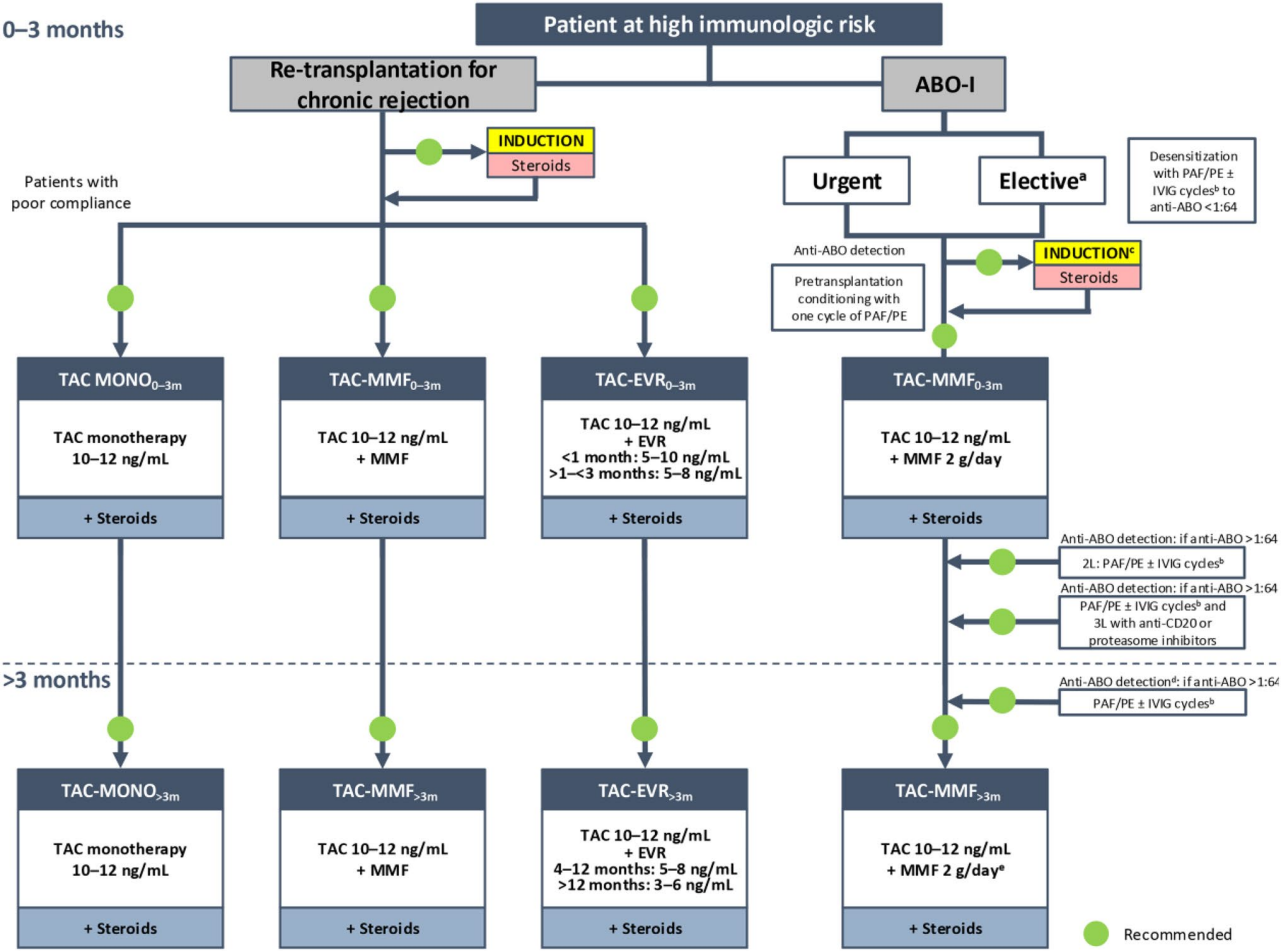
Algorithm for immunosuppressive therapy in patients at high immunologic risk undergoing liver transplantation. The indicated target blood levels of immunosuppressants are not binding. Patients with chronic graft rejection should first receive induction therapy (basiliximab) plus corticosteroids followed by tacrolimus in monotherapy plus corticosteroids for patients with compliance problems, or CNI-reducing regimens with tacrolimus and mycophenolate mofetil or everolimus, plus corticosteroids in both cases. In ABO-I patients, transplant surgery can be urgent or elective. Candidates for elective surgery should be desensitized with cycles of plasmapheresis/plasma exchange with or without immunoglobulins to reach an anti-ABO titer < 1:64. Patients needing an emergency intervention should be conditioned preoperatively with one cycle of plasmapheresis/plasma exchange. Following conditioning, the immunosuppression protocols for emergency and elective liver transplants are similar and involve induction therapy plus corticosteroids followed by tacrolimus–mycophenolate mofetil with corticosteroids. Monitoring of anti-ABO titer is recommended; if the titer is > 1:64, second-line immunomodulatory treatment involves cycles of plasmapheresis/plasma exchange with or without immunoglobulins. If the ABO titer continues to be > 1:64, third-line treatment is recommended with use of antiCD20 agents or proteosome inhibitors. At > 3 months post-transplantation, the recommended regimens remain the same as during the 3 months following the intervention, with a decrease in the tacrolimus dose. ^a^Organ from a living donor. ^b^Frequency and duration of PAF/PE depend on anti-A and anti-B titers. ^c^The use of polyclonal antibodies (ALG, ALT) requires monitoring and caution due to the high risk of infections and related complications. ^d^Repeat weekly during the first month. ^e^Recommended dose (may vary depending on individual center protocols). Adjustments of immunosuppressive therapy should be made based on patient clinical characteristics. *2L* second line, *3L* third line, *ABO-I ABO* incompatible, *ALG* antilymphocyte globulin, *ALT* alanine aminotransferase, *CNI* calcineurin inhibitor, *EVR* everolimus, *IVIG* intravenous immunoglobulin, *MMF* mycophenolate mofetil, *MONO* monotherapy, *PAF* plasmapheresis, *PE* plasma exchange, *TAC* tacrolimus

The original article has been corrected.

