# Supplementary material for: Immunosuppression in adult liver transplant recipients: a 2024 update from the Italian Liver Transplant Working Group
Source: Hepatol Int. 2024 Jul 15;18(5):1416–30. doi: 10.1007/s12072-024-10703-4 (PMC11461624; doi:10.1007/s12072-024-10703-4)
Supplement: Supplementary file 1 — Supplementary file1 (DOCX 150 KB) [file 12072_2024_10703_MOESM1_ESM.docx]

**Immunosuppression in adult liver transplant recipients: a 2022 update from the Italian Liver Transplant Working Group**

**Authors:** Tommaso Maria Manzia^1^, Barbara Antonelli^2^, Amedeo Carraro^3^, Grazia Conte^4^, Nicola Guglielmo^5^, Andrea Lauterio^6^, Laura Mameli^7^, Umberto Cillo^8^, Luciano De Carlis^9,10^, Massimo Del Gaudio^11^, Paolo De Simone^12^, Stefano Fagiuoli^13^, Francesco Lupo^14^, Giuseppe Tisone^1^, Riccardo Volpes^15^ on behalf of the Italian Liver Transplant Working Group

**Affiliations:** ^1^Department of Surgical Science, University of Rome Tor Vergata, Rome, Italy; ^2^Fondazione IRCCS Ca’ Granda Ospedale Maggiore Policlinico, Milan, Italy; ^3^Liver Transplant Unit, University Hospital Trust of Verona, Verona, Italy; ^4^Clinica di Chirurgia Epatobiliare, Pancreatica e dei Trapianti, Azienda Ospedaliera Universitaria delle Marche, Ancona, Italy; ^5^General Surgery and Liver Transplantation Unit, Azienda Ospedaliera San Camillo-Forlanini, Rome, Italy; ^6^ASST Grande Ospedale Metropolitano Niguarda; University of Milano-Bicocca, Milan, Italy; ^7^Azienda Ospedaliera G. Brotzu, Cagliari, Italy; ^8^Hepatobiliary and Liver Transplant Unit, University Hospital of Padua, Padua, Italy; ^9^Department of General Surgery and Transplantation, Niguarda Hospital, Milan, Italy; ^10^School of Medicine, University of Milano-Bicocca, Milan, Italy; ^11^Department of General Surgery and Transplantation, Policlinico S. Orsola-Malpighi, Bologna, Italy; ^12^Hepatobiliary Surgery and Liver Transplantation Unit, University of Pisa Medical School Hospital, Pisa, Italy; ^13^Gastroenterology, Department of Medicine, University of Milano-Bicocca and Gastroenterology Hepatology and Transplantation, Papa Giovanni XXIII Hospital, Bergamo, Italy; ^14^Department of General Surgery, Azienda Ospedaliera Città della Salute e della Scienza, Turin, Italy; ^15^Mediterranean Institute for Transplantation and Advanced Specialized Therapies (ISMETT/IRCCS), Palermo, Italy; Fondazione Istituto G. Giglio di Cefalù (Palermo), Italy.

**Corresponding author:** Prof. Stefano Fagiuoli, Gastroenterology Hepatology and Transplantation, Papa Giovanni XXIII Hospital, Piazza OMS, 124127 Bergamo, Italy. Email: [sfagiuoli@asst-pg23.it](mailto:sfagiuoli@asst-pg23.it)

# Supplementary material

# Supplementary material

## Supplementary methods

Critical patients were defined as those with any of the following conditions: 1) national high-urgency status; 2) requiring intensive care unit (ICU) and/or ventilatory support and/or inotropic support; 3) recent extrahepatic sepsis or ongoing liver sepsis/spontaneous bacterial peritonitis/multidrug-resistant organism-colonized; portal thrombosis III-IV; 4) thoracic ascites; 5) sarcopenia (i.e., either Child C or Child A or B with body mass index <18.5 kg/m^2^, psoas muscle area at L3 <50 cm^2^/m^2^ in males or <39 cm^2^/m^2^ in females); 6) frailty (i.e., Short Physical Performance Battery score <10); 7) high Model for End-stage Liver Disease-sodium (MELD-Na) score (i.e., ≥29 or 25–29 in patients with renal dysfunction/dialysis or chronic encephalopathy); 8) acute gastroesophageal bleeding; 9) hepatorenal syndrome; 10) high surgical risk (i.e., previous abdominal surgery, retransplantation, or complex surgical procedure); 11) renal dysfunction at transplantation (i.e., estimated glomerular filtration rate [eGFR] <90 mL/min/1.73 m^2^, proteinuria >0.5 g/24 h, MELD-Na ≥21, refractory ascites, national high-urgency status) or post-transplantation (i.e., eGFR <90 mL/min/1.73 m^2^, proteinuria >0.5 g/24h, eGFR slope decline ≥4 mL/min/1.73 m^2^/year); 12) organ donated after circulatory death with post reperfusion problems or delayed graft function; 13) metabolic syndrome/non-alcoholic steatohepatitis based on the National Cholesterol Education Program-Adult Treatment Panel (NCEP-ATP) III 2005 criteria for metabolic syndrome *(i.e., the presence of ≥3 of the following: i) abdominal obesity [waist circumference >102 cm in men or >88 cm in women], ii) high triglycerides [>150 mg/dL] or on medication for high triglycerides, iii) low high-density lipoprotein-cholesterol [<40 mg/dL in men or <50 mg/dL in women] or on lipid-lowering medication, iv) arterial hypertension [>130 mmHg systolic blood pressure and >85 mmHg diastolic blood pressure] or on antihypertensive medication, v) high fasting blood glucose [>100 mg/dL] or on specific medication or previous diagnosis of type 2 diabetes mellitus)*.

It is important to consider induction therapy for the management of critical patients in the first 3 months following LT. Induction is indicated to allow delayed CNI introduction, early CNI minimization, and a steroid-free approach. Patients with an infection contracted after the transplant procedure should be considered for reduction or discontinuation of the immunosuppressive treatment.

## Supplementary Fig. 1

Algorithm for immunosuppressive therapy in standard patients undergoing liver transplantation. The indicated target blood levels of immunosuppressants are not binding. Early reduction of CNIs (tacrolimus) and addition of mycophenolate mofetil or everolimus is recommended; induction therapy with basiliximab to postpone CNI use may be possible. With some caution, a CNI-free immunosuppressive regimen based on everolimus alone may be considered, both at 0–3 months and >3 months post-transplantation. Overall, monotherapy with a CNI is not recommended and the immunosuppressive regimen should preferably be steroid-free.


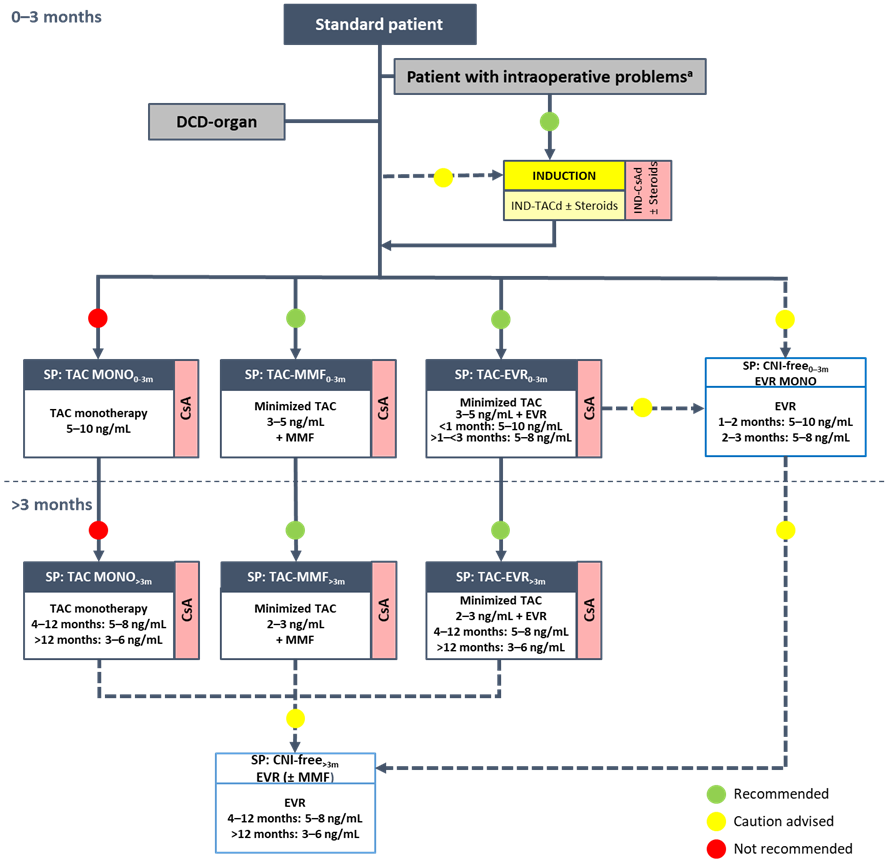


^a^If a severe intraoperative event occurs, consider switching to “Critical Patient – High surgical risk”. *CNI* calcineurin inhibitor, *CsA* ciclosporin, *d* delayed, *DCD* donated after circulatory death, *EVR* everolimus, *IND* induction, *MMF* mycophenolate mofetil, *MONO* monotherapy, *SP* standard patient, *TAC* tacrolimus.
